# Biological Approaches Integrating Algae and Bacteria for the Degradation of Wastewater Contaminants—A Review

**DOI:** 10.3389/fmicb.2021.801051

**Published:** 2022-02-03

**Authors:** Merwin Mammen Mathew, Kanchan Khatana, Vaidehi Vats, Raunak Dhanker, Ram Kumar, Hans-Uwe Dahms, Jiang-Shiou Hwang

**Affiliations:** ^1^Department of Basic and Applied Sciences, School of Engineering Sciences, GD Goenka University, Gurugram, India; ^2^Ecosystem Research Laboratory, Department of Environmental Science, School of Earth, Biological and Environmental Sciences, Central University of South Bihar, Fatehpur, India; ^3^Department of Biomedical Science and Environmental Biology, Kaohsiung Medical University, Kaohsiung, Taiwan; ^4^Institute of Marine Biology, National Taiwan Ocean University, Keelung, Taiwan; ^5^Center of Excellence for Ocean Engineering, National Taiwan Ocean University, Keelung, Taiwan; ^6^Center of Excellence for the Oceans, National Taiwan Ocean University, Keelung, Taiwan

**Keywords:** microalgae, bacteria, wastewater treatment, integrated approach, sustainable method

## Abstract

The traditional approach for biodegradation of organic matter in sewage treatment used a consortium of bacterial spp. that produce untreated or partially treated inorganic contaminants resulting in large amounts of poor-quality sludge. The aeration process of activated sludge treatment requires high energy. So, a sustainable technique for sewage treatment that could produce less amount of sludge and less energy demanding is required for various developed and developing countries. This led to research into using microalgae for wastewater treatment as they reduce concentrations of nutrients like inorganic nitrates and phosphates from the sewage water, hence reducing the associated chemical oxygen demand (COD). The presence of microalgae removes nutrient concentration in water resulting in reduction of chemical oxygen demand (COD) and toxic heavy metals like Al, Ni, and Cu. Their growth also offers opportunity to produce biofuels and bioproducts from algal biomass. To optimize use of microalgae, technologies like high-rate algal ponds (HRAPs) have been developed, that typically use 22% of the electricity used in Sequencing Batch Reactors for activated sludge treatment with added economic and environmental benefits like reduced comparative operation cost per cubic meter, mitigate global warming, and eutrophication potentials. The addition of suitable bacterial species may further enhance the treatment potential in the wastewater medium as the inorganic nutrients are assimilated into the algal biomass, while the organic nutrients are utilized by bacteria. Further, the mutual exchange of CO_2_ and O_2_ between the algae and the bacteria helps in enhancing the photosynthetic activity of algae and oxidation by bacteria leading to a higher overall nutrient removal efficiency. Even negative interactions between algae and bacteria mediated by various secondary metabolites (phycotoxins) have proven beneficial as it controls the algal bloom in the eutrophic water bodies. Herein, we attempt to review various opportunities and limitations of using a combination of microalgae and bacteria in wastewater treatment method toward cost effective, eco-friendly, and sustainable method of sewage treatment.

## Introduction

Bacteria and algae are extensively used in the treatment of wastewater (Almomani et al., 2019). The parameters that influence the growth of microbes are firstly geographical location, secondly the type of pond in which bacteria will be grown, thirdly the characteristics of the wastewater entering the plant, and finally the operating parameters of the system, such as aeration, agitation, and chemical injection. All of these factors make quantitative changes between autotrophic and heterotrophic bacteria (Marcin et al., 2018). Gram-negative bacteria of the proteobacteria type are a predominant group of bacteria of which Betaproteobacteria is the most abundant class which is largely responsible for the elimination of organic elements and nutrients. The other branches are Bacteroidetes, Acidobacteria, and Chloroflexi. The most numerous types of bacteria are *Tetrasphaera*, *Trichococcus*, *Candidatus Microthrix*, *Rhodoferax*, *Rhodobacter*, and *Hyphomicrobium* that are useful for the degradation of microbes (Gunther et al., 2020).

Microalgae culture offers an economically viable and eco-friendly method for wastewater treatment, because they provide a tertiary biotreatment coupled with the production of valuable biomass, which can be used for several purposes (Alexandre et al., 2018; Gudiukaite et al., 2021). Microalgae cultures offer an elegant solution to tertiary and quaternary treatments due to the ability of microalgae to use inorganic nitrogen and phosphorus for their growth. They are also known for their capacity to remove heavy metals and some toxic organic compounds, thus can also help to prevent secondary pollution (Raouf et al., 2012; Arora et al., 2021; Dhanker et al., 2021).

Microalgae and bacteria are used to recover nutrients from wastewater as alternative to conventional technologies such as those based on activated sludge. Since no sterile conditions are possible in wastewater systems, the naturally occurring bacterial consortium prevails in the reactors and its occurrence is a function of the wastewater composition, environmental conditions, reactor design, and operation conditions (Muñoz and Guieysse, 2006). Bacteria utilize the organic wastes present and produce CO_2_ as a by-product. The microalgae in turn utilize this CO_2_ to produce carbohydrates and O_2_ through photosynthesis, of which the former is needed for biomass production, while the latter serves as the terminal electron acceptor for aerobic respiration in bacteria. According to this scheme, a “natural” equilibrium is established between microalgae and bacteria based on the conditions of the reactor. However, the composition of the consortium in this equilibrium can differ widely depending on the conditions prevailing in the reactor. The consortium composition directly affects the ratios between the various phenomena like oxygen production, CO_2_ consumption, nitrogen, and phosphorus assimilation. Therefore, the levels of these processes keep changing alongside the change in consortia (Molazadeh et al., 2019). Wastewater treatment efficiency of microorganisms can be improved by eliminating the fats and oils responsible for the habitat degradation by manipulating the presence of appropriate bacterial species for the treatment of cold or hot water (Zahara et al., 2020).

This paper begins with a succinct discussion on the need of wastewater treatment and then presents a glimpse into the current trends in the independent use of bacteria and microalgae for wastewater treatment, and finally explains the integrated application of microalgae and heterotrophic bacteria for eco-friendly sewage treatment process based on researches done in the area.

## Relevance of Wastewater Treatment in Current Scenario

The modern-day aerobic sewage treatment plants make use of natural air currents. This process eliminates the organic pollutants and their associated odor by providing conditions for their complete oxidation into carbon dioxide, nitrogen, and water. The treated effluent is thus pollutant free and can be discharged for use (Marina et al., 2019). Water treatment facilities are designed to speed up the natural process of purifying water. With billions of people, the natural process is overloaded. Without proper wastewater treatment, the amount of wastewater would cause devastation, as it still does today in developing countries. Globally, over 80% of all wastewater is discharged without treatment. Countries having water treatment facilities use various methods to treat wastewater with one common goal, i.e., purify water as much as possible and send it back into the environment to keep humans and the Earth safe and thriving (Holger et al., 2006; Molazadeh et al., 2019).

Wastewater contains elements which are toxic to humans and the ecosystem. Wastewater treatment facilities help to purify the water and improve the situations like what is currently seen in developing countries (Bjorn et al., 2006; Desai et al., 2021). Untreated water poses significant health risks, accounting for 1.7 million deaths annually, of which over 90% are in developing countries. Several water-related diseases, including cholera and schistosomiasis, remain widespread across many developing countries, where only a very small fraction of domestic and urban wastewater is treated prior to its release into the environment (Richard, 2019). Presence of heavy metal in water results in severe health issues including cardiovascular disorders, neuronal damage, renal injuries, and risk of cancer and diabetes (Jaishankar et al., 2014). While Mother Nature does her best to naturally process wastewater, there is too much for her to handle. The amount of wastewater is increasing day by day in tune with the global population increase (Chia et al., 2007; Cristine et al., 2011).

## Current Wastewater Treatment

**FIGURE 1:**
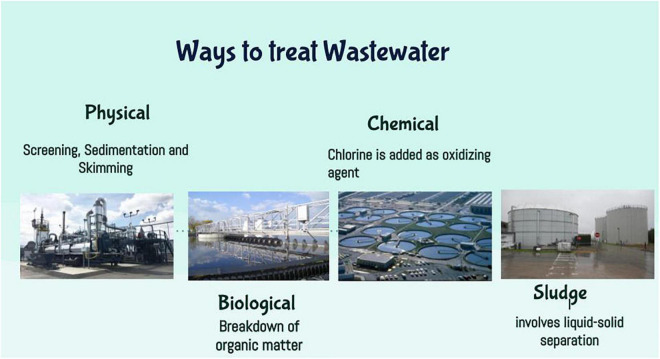
Ways to treat wastewater.

Four common ways to treat wastewater include physical water treatment, biological water treatment, chemical treatment, and sludge treatment (Figure 1).

### Physical (Conventional) Water Treatment

Processes like screening, sedimentation, and skimming are used to remove the solids. No chemicals are involved in this process. One of the main techniques of physical wastewater treatment includes sedimentation, i.e., a process of suspending the insoluble or heavy particles from the wastewater. Separation of pure water can be done when the insoluble material settles down at the bottom (Mathew and Alan, 2009). Another effective physical water treatment technique includes aeration. This process consists of circulating air through the water to provide oxygen to it. Filtration, the third method, is used for filtering out all the contaminants. Special custom designed filters are used to separate the contaminants and insoluble particles by passing the wastewater through the filter. The sand filter is the most commonly used filter. The grease found on the surface of some wastewater can also be removed easily through this method (Harleman and Murcott, 1999).

### Biological Water Treatment

This uses various biological processes to break down the organic matter present in wastewater, such as soap, human waste, oils, and food. Microorganisms metabolize organic matter in the wastewater in biological treatment. It can be divided into three categories: (a) aerobic processes: bacteria decompose organic matter by using oxygen as the oxidizing agent, leading to its conversion to carbon dioxide that can then be used by plants; (b) anaerobic processes: here, fermentation is used for fermenting the waste at a specific temperature. Oxygen is not used in anaerobic process, and (c) composting: a type of aerobic process where wastewater is treated by mixing it with sawdust or other carbon sources (Yuansong et al., 2003; Arun, 2011).

### Chemical Water Treatment

This treatment involves the application of chemicals in water. Chlorine, an oxidizing chemical, is commonly used to kill bacteria which decomposes water by adding contaminants to it (Jari et al., 2003). Another oxidizing agent used for purifying the wastewater is ozone. Neutralization is a technique where an acid or base is added to bring the water to its natural pH of 7. Chemicals prevent the bacteria from reproducing in water, thus making the water pure (Ødegaard and Karlsson, 1994).

### Sludge Treatment

This is a solid-liquid separation process where the least possible residual moisture is required in the solid phase and the lowest possible solid particle residues are required in the separated liquid phase (Tong et al., 1980; Krist et al., 2004).

## The Role of Bacteria and Microalgae in Wastewater Treatment

### Microalgal Treatment

Microalgae include prokaryotic cyanobacteria and eukaryotic autotrophic protist-algae that are too small to be seen without a microscope (Larsdotter, 2006). Microalgae are primary producers of any aquatic ecosystem which are the main food of secondary producers (Dhanker et al., 2012, 2013). Algal cultures are being used for the treatment of wastewater from last 75 years, and today the biologists in United States, Thailand, Taiwan, and Mexico are taking substantial interest in this area due to their understanding of the biology, ecology, and engineering of large-scale algal culture systems (Raouf et al., 2012). Many factors like fear of food insecurity, rise of property cost, and consciousness toward the need for environmental protection have greatly promoted the development of microalgal water treatment systems (Paddock, 2019).

Microalgae purify water through either direct uptake of contaminants or their transformation to harmless products (Dhanker et al., 2021). Algal species are reported to facilitate bacterial degradation of organics by providing oxygen through photosynthesis, which reduces costs and energy expenditure associated with the gassing/stirring process for aeration (Wollmann et al., 2019). Nitrogen, phosphorous, and carbonaceous substances are taken up by microalgae to build up their biomass. The nitrogen assimilated is in the form of ammonia, nitrate, and nitrite; at the same time, carbon dioxide removal is also possible as it is a photosynthetic substrate. Additionally, microalgae have potential to remove heavy metals from water (Ahmed et al., 2022) and may be utilized for production of biofuel source, due to a much higher per unit area yield than terrestrial oil seed crops (Wang et al., 2010; Gudiukaite et al., 2021).

The mode of CO_2_ fixation employed by microalgae is called oxygenic photosynthesis which transforms it to reduced carbon compounds like sugars in the presence of water and light (Benedetti et al., 2018). This process is mediated by four membrane protein complexes that are multi-subunit in nature, namely, photosystem 1, photosystem 2, cytochrome b_6_f, and F-ATPase. These complexes are found on the thylakoid membranes of chloroplast and the equivalent structures of cyanobacteria and carry out the light dependent oxidation of water to oxygen, reduction of NADPH (nicotine adenine dinucleotide phosphate), and the synthesis of ATP (adenosine triphosphate). The two photosystems separately oxidize water and reduce NADPH, and the cytochrome transfers electrons between the photosystems while also generating the PMF (proton motive force) for ATP synthesis which in turn is the job of F-ATPase. In the dark, the stroma of the chloroplast acts as the site of the Calvin cycle that takes in CO_2_ (carbon source) from the environment and the ATP (energy source) and NADPH (reducing agent) from the light reaction to produce carbohydrates (Nelson and Shem, 2004). The photosynthetically active radiation (PAR) ranges between 400 and 700 nm, and 8 such PAR photons of light at 550 nm are required for CO_2_ fixation or splitting of 2 water molecules to one molecule of diatomic oxygen. Practically, this becomes 10 photons as photosynthesis is not 100% efficient (Su, 2021). The relative CO_2_ fixation concentrations of different microalgae species were evaluated and found to be highest in *Synechocystis aquatilis*—1500 mg/L/day, *Botryococcus braunii*—1,100 mg/L/day, *Chlorella vulgaris*—865 mg/L/day, and in *Synechococcus* spp. Species that fixed higher CO_2_ concentrations were also found to generate higher biomass, in general (Singh and Singh, 2014).

Nitrogen is an essential component making up to 1–10% (6–10% in case of *Chlorella*) of a cell’s dry in the form of its building blocks like the genetic material, enzymes, proteins, vitamins, hormones, alkaloids, energy transfer molecules, and amides (Jia and Yuan, 2016). Ammonium (NH_4_^+^) is preferred as a nitrogen source in green algae as assimilation of other sources is partially or completely inhibited in its presence. It is assimilated by the help of the glutamine synthase (GS)–glutamate synthase cycle. Glutamate synthase is also called glutamine oxoglutarate aminotransferase or GOGAT. Some green algae under certain conditions use the NADP-glutamate dehydrogenase (GDH) pathway for its assimilation. Algae like *Tetraselmis striata* have high levels of GS and GOGAT indicating its dominant role in nitrogen assimilation in them, over GDH whose levels are lower (Hellebust and Ahmad, 1989). Still at concentrations above 100 mg/L, ammonium proves to be toxic to microalgae as it converts to ammonia that inhibits microalgal photosynthesis (Su, 2021). The other nitrogen sources being important for algal growth, generally present in natural waters with higher concentrations, are nitrate and nitrite. Their assimilation is dependent on the activity and capacity of nitrate and nitrite transport systems of cells, then the amount and activity of cellular nitrate and nitrite reductase. The genes of nitrate and nitrite reductase are repressed by the presence of ammonia, and for green algae grown on reduced nitrogen sources, presence of nitrate and absence of reduced nitrogen are essential for the induction of nitrate reductase. The final source is the organic nitrogen as in amino acids or urea. This source is variable from species to species as it is dependent on the differences in transport abilities and degradation enzymes. For instance, *Stichococcus bacillaris* possess systems for the active transport of multiple acidic, basic, and neutral amino acids, while some species of the order Volvocales have active transport systems for arginine but cannot transport other amino acids (Hellebust and Ahmad, 1989).

For heavy metal removal from water, microalgae employ the following three mechanisms, i.e., extracellular precipitation that needs living cells, cell surface sorption/complexation which can be done by both living or dead cells, and intracellular accumulation which brings in the need for metabolic processes of the microalgae. Microalgae species like *Chlamydomonas reinhardtii* contribute efficiently to the removal of Cadmium, and live cells of *Planothidium lanceolatum* take up large quantities of Cd^2+^ at 275.51 mg/g. For Cobalt removal *Spirogyra* spp. and *Oscillatoria angustissima* prove to be promising. *Chlorella* and *Spirulina* spp. are good at removing hexavalent Chromium (Cr^6+^) with *Spirulina* having a chromium uptake rate of 333 mg/g. *Spirulina* species has been shown to remove copper at 389 mg/g along with nickel and zinc, both at 1,378 mg/g. All these remediation activities take place under different ranges of pH (Kumar et al., 2015).

Phosphorus is used by microalgae to produce nucleic acids-DNA (deoxyribonucleic acid) and RNA (ribonucleic acid), ATP for energy, and phospholipids of the membrane. The most preferred forms for uptake are PO_4_^3–^, HPO_4_^2–^, H_2_PO_4_^–^ of which the ones with lower charge have greater bioavailability, but polyphosphate is also available for use in microalgae. Phosphite (PO_3_^3–^) utilization has only been demonstrated in cyanobacteria. Inorganic phosphate is taken inside the cell *via* Pi transporters, while phosphatases on the membrane surface hydrolyze phosphate containing organic compounds and import the phosphate released. The phosphate assimilated in the cells is elongated upon to produce acid soluble polyphosphate (ASP) or acid insoluble polyphosphate (AISP) by polyphosphate kinase by the hydrolysis of ATP to ADP (adenosine diphosphate). High light intensity promotes formation of ASP and its conversion to proteins and DNA. The extracellular phosphorous compounds after internalization are converted to cellular phosphate that is used for synthesis of phospholipids and RNA, though the transfer of phosphate to RNA is inhibited by light. When excess phosphorus is found in the environment, or if the cell is transferred from a deficient to rich environment with respect to phosphorus, then it assimilates more phosphorus than what is needed for survival. This excess phosphate assimilated is largely transformed to AISP and stored in vacuoles for use in case of future phosphorus deprivation, by transfer to other algal phosphorus containing compounds for cell viability (Su, 2021). The microalgae *C. vulgaris* is commonly used for removal of N, P compounds and heavy metals from the wastewater during tertiary treatment (de-Bashan et al., 2002). The capacities of microalgae to remove nitrogen and phosphorus from different wastewater sources can be seen in Table 1, for example, *Coelastrum microporum* that removes 88 and 89% of the total nitrogen and phosphorus contents, respectively.

**TABLE 1:** Showing effectiveness of different microalgal species at removing total nitrogen (TN) and total phosphorus (TP) from different wastewaters.

Main microalgae	Wastewater used	TN initial concentration (mg/L)	TN removed (%)	TP initial concentration (mg/L)	TP removed (%)	References
*Micractinium inermum NLP-F014*	Bold modified basal freshwater (BMBF) nutrient solution	36 ± 1.1	95.69	49 ± 0.71	10.71	Jeong and Jang, 2020
*Scenedesmus dimorphus*	Dairy industry wastewater	36.3	>90	112	20–55	Salces et al., 2019
*Micractinium reisseri*	Piggery wastewater	53	7.547	7.1	2.817	Abou-Shanab et al., 2013
*Chlamydomonas* sp. (YG04)	Municipal wastewater secondary effluent	190.7 ± 0.12	77.57	19.11 ± 0.03	100	Amini et al., 2014
*Nitzschia* cf. *pusilla*	Piggery wastewater	53	15.09	7.1	9.859	Abou-Shanab et al., 2013
*Coelastrum microporum*	Municipal wastewater	40	88	5.3	89	Salces et al., 2019
*Chlorella zofingiensis*	Untreated and undiluted pig anaerobic digested effluent	1011–1050	82.7	25–26.5	98.17	Li et al., 2019
*Mucidosphaerium pulchellum*	Domestic wastewater	64–79	79	4.6–7.2	49	Salces et al., 2019

Many microalgae species exhibit a mixotrophic mode of nutrition based on light conditions, due to which they can switch between auto and heterotrophy. This allows them to use organic carbon as biomass, which then contributes to greater biomass removal during wastewater treatment. Still, they lack a proper organic carbon uptake mechanism and transport pathway due to which only a few of them can grow heterotrophically like *C. reinhardtii*. Organic carbon sources are limited to sugars (galactose, glucose, to some extent fructose), alcohols (glycerol and ethanol), and acids (acetic acid). Glucose is metabolized *via* the Embden Meyerhof Pathway (EMP) under lighted conditions or by pentose phosphate pathway (PPP) in the dark heterotrophically. The resulting pyruvate forms acetyl CoA and undergoes the TCA (tricarboxylic acid) cycle under aerobic conditions (Su, 2021).

#### The Factors Affecting the Treatment of Wastewater by Microalgae

The factors affecting the treatment of wastewater by microalgae which are often closely associated with their growth are:

(a) CO_2_ availability, which may reduce the developmental rate of algal biomass if found to be low. Hence, the rate of nutrient and heavy metal assimilation should also decrease. For optimum algal growth air enriched with 1–5% CO_2_ may be provided as atmospheric CO_2_ levels are far below optimum at 0.033% (Larsdotter, 2006).
(b) High ammonium ion concentrations of water (100 mg/L) cause ammonia to form that inhibit the growth of algae at concentrations >30 mg/L and pH 9 (Park and Craggs, 2011), by obstructing the microalgal photosynthetic pathway (Su, 2021).
(c) The higher pH level reduces CO_2_ uptake, that interferes with the activity of the RuBisCO enzyme, and promotes the dissociation of NH_4_^+^ to NH_3_ (Salces et al., 2019).
(d) O_2_ concentrations higher than 20 mg/L causes photorespiration and O_2_ radical formation resulting in partial inhibition of photosynthesis which together leads to decreased microalgal growth (de Godos et al., 2017).
(e) Temperature, which causes growth to increase up to a certain point, but which declines rapidly beyond this threshold (Larsdotter, 2006). Though temperature is strain dependent, its optimum generally lies between 20 and 30°C and maintaining cultures at these optimum temperature’s leads to greater nutrient removal efficiencies (Salces et al., 2019).
(f) Light increases the growth of microalgae due to increasing photosynthesis rates up to a certain threshold after which it decreases photosynthetic rates (Park et al., 2011).
(g) Cynobacterial inhibitory substances reduce the growth of eukaryotic algae (Larsdotter, 2006).
(h) Presence of parasites and algae virus organisms, e.g., rotifers and protozoa, interferes with the rate at which algae treats wastewater and produces important by-products (Grobbelaar, 1982; Mostert and Grobbelaar, 1987).
(i) Presence of acetate which is toxic to some species as its un-ionized form can penetrate the cell membrane and enter inside and ionize there, thus causing internal damage (Larsdotter, 2006).
(j) High quantities of organic substances inhibit nutrient uptake by microalgae (Ogbonna et al., 2000).

#### Wastewater Treatment Using High-Rate Algal Pond: A Microalgal Technology

**FIGURE 2:**
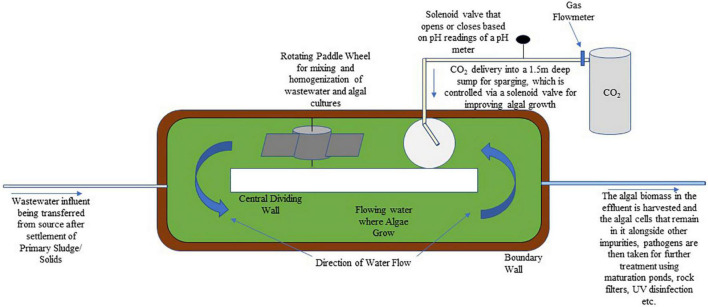
A schematic showing a high-rate algal pond that has a CO_2_ addition function for improving algal growth.

High-rate algal pond (HRAP) is a technology developed in the 1950s that uses microalgae for resource recovery and treatment of wastewater. It is an open raceway pond that uses a paddlewheel for mixing water, while also having a higher pathogen treatment and nutrient removal efficiency over traditional wastewater treatment systems. To make the process even more economically feasible, the microalgal biomass that develops can be used as protein-rich feed, fertilizer, and most importantly for biofuel production (Sutherland et al., 2014). Biofuel production using HRAPs is popular as the extra costs of algal cultivation and biodiesel production associated with nutrient supply are taken care of by the nutrients present in wastewater or by the microalgal function of wastewater treatment (Mehrabadi et al., 2015). As it relies on algal photosynthesis, it operates best in arid, semi-arid, or tropical climates, and has been used for the treatment of wastewaters from domestic, tannery, dairy, and piggery sources. Removal of nitrogen is attributed to its assimilation into algal biomass and ammonia volatilization. Nitrogen removal in HRAPs is between 26.6 and 75.7% with 61.23% median, and ammonia removal is between 21.89 and 94% with a 77% median. Phosphorus in HRAPs on the other hand is removed by assimilation into biomass and by its pH dependent precipitation. Its removal range is around 10.48–97.2% with a 42.73% median (Young et al., 2017). The High-Rate Algal Pond (HRAP) technology has been developed that has a CO_2_ addition function for improving algal growth (Figure 2).

In India, according to a report by the Central Pollution Control Board, New Delhi, only 20–30% of the 40 billion liters of wastewater generated is treated. This means like in many parts of the world the need for clean water and sustainable energy providing sources is of prime importance in India. Still algal growth on wastewater in HRAPs could only achieve 0.16 Mt/annum of biofuel production, if the lipid fraction of algal cells is approximated to be 20%. So even though HRAPs achieve high biomass contents, lipid productivities, and nutrient removal efficiencies, they do not compete well with other fuels whose use is still more economical and sustainable as per today’s energy prices (Bhatt et al., 2014).

### Bacterial Treatment

Bacteria are a critical requirement for the wastewater treatment strategy that was developed in the early 20th century and now serving as the principal of water treatment worldwide. Bacteria, some protozoans, and other microbes are confined to a tank in higher concentrations utilizing organic carbon as nutrient source. Therefore, the organic carbon in wastewater act as a stimulant of bacterial growth leading to natural purification prior to discharge. The microorganisms that treat the wastewater are collectively referred to as activated sludge (Davies, 2005).

The removal of nitrogen from wastewater involves three main processes, which are nitrification, denitrification, and anaerobic ammonia oxidation (anammox). In the nitrification process, aerobic chemo-lithoautotrophic ammonia-oxidizing bacteria (AOB) and nitrite oxidizing bacteria (NOB) catalyze the two-step process in the given sequence respectively. The main ammonia oxidizer found in many systems is *Nitrosococcus mobilis* and the most important NOB in treatment plants are the bacteria of the genus *Nitrosospira* (Daims et al., 2006). Denitrification can either be a heterotrophic or an autotrophic process. In the heterotrophic route nitrate reduction following uptake is carried out using electron donors in the form of carbonaceous organic matter. Different species show differences in nitrogen metabolism when using different carbon sources, as in *Anoxybacillus contaminans* is shown to carry out both nitrification and aerobic denitrification in the presence of glucose, while *Zoologea* spp. showed 78.86% nitrate removal with sodium acetate as substrate (Rajta et al., 2020). Autotrophic denitrification is carried out by Sulfur oxidizing bacteria (SOB) that are capable Sulfur oxidizing autotrophic denitrification (SOAD) which uses reduced compounds like thiosulphate, sulfide, and elemental sulfur for reducing nitrate and nitrite. These reactions can be mediated by Proteobacteria of the genera *Thiobacillus*, *Paracoccus*, *Sulfurimonas*, and *Thioalkalivibrio* (Cui et al., 2019). In case of anammox, nitrite and ammonia combine to produce dinitrogen gas, and this requires aeration in the initial phase for the partial oxidation of ammonia to nitrite. Inhibition of NOB that uses nitrite as a substrate is also necessary. It removes nitrogen without addition of any extra organic carbon hence is suitable for high ammonia and low carbon content effluents. The bacteria that carry it out are members of the genus Planctomycetes which are chemolithoautotrophic, though they have slow growth rates and long build uptime in the initial working phase of anammox reactors (Daims et al., 2006). Nitrate reduction is carried out using assimilatory, dissimilatory, and denitrification pathways. Assimilatory pathways convert it to biomass, dissimilatory pathway to ammonium, and denitrification converts it to dinitrogen (Rajta et al., 2020).

The bacteria mediated biological removal of phosphorus from water using bacteria is achieved through the process of enhanced biological phosphorus removal (EBPR). This process exploits the ability of bacteria to accumulate polyphosphate inside their cells and selects for them using sequential aerobic and anaerobic phases. It uses two classes of bacteria, phosphorus accumulating organisms (PAO) and glycogen accumulating organisms (GAO). This process is at times unreliable as these two classes compete for carbon, hence leading to EBPR breakdowns. To avoid this, stimulating the growth of actinobacterial PAO over others is thought to help (Daims et al., 2006).

Organic matter present in wastewater is hydrolyzed by bacterial cells through the means of extracellular enzymatic hydrolysis as their cell walls prevent the process of phagocytosis. This is the case with particles in the size range 10^3^ amu–100 μm. Particles smaller than this can be directly taken up by cells and the ones larger are removed by sedimentation. Degradation processes are primarily de-polymerization reactions that are mostly performed through hydrolytic enzymes and in fewer cases through lyases. Exo-enzymes depolymerize at the ends that are usually the non-reducing ones, while endo-enzymes depolymerize at the terminal monomers (Morgenroth et al., 2002). Once inside the cell, simpler compounds are used directly during feast period and the polymers formed during feast period are degraded to release simpler organic compounds in heterotrophic bacteria during times of starvation. This energy formed from catabolism is then used to fuel life processes like motility, ion gradient maintenance, transport of materials, and protein/nucleic acid turnover (Hao et al., 2010). Biological nutrient removal (BNR) is a function of bacterial metabolism and/or bacterial growth; hence, choosing species with higher rates of these two is ideal for this purpose (Pollard and Greenfield, 1997).

#### The Factors Affecting the Treatment of Wastewater by Bacteria

The factors affecting the treatment of wastewater by bacteria are:

(a) Anammox bacteria are found to be inactivated/eradicated at organic matter concentrations higher than 300 mg COD/L where the COD to nitrogen ratio is 2, if anammox and denitrification processes happen side-side. This happens as they are unable to compete with denitrifying bacteria (Ni et al., 2012).
(b) Methanol concentration of 0.5 mM is also found to completely and immediately inhibit anammox activity (Ni et al., 2012).
(c) The change of the organic compound composition changes from volatile fatty acids to sugars triggers the accumulation of Glycogen (Mulkerrins et al., 2004). These as stated before compete with Phosphate accumulating organisms hence causing EBPR breakdown (Daims et al., 2006).
(d) The co-transport of potassium and magnesium alongside phosphorus help in the stabilization of intracellular polyphosphate (Rickard and McClintock, 1992).
(e) Temperature affects microbial activity, gas transfer rates, and setting properties of biological solids. Every time temperature is increased by 10°C, growth rates double. This means nutrient removal also increases, for example, at higher temperatures of 20–37°C, biological phosphorus removal efficiency is said to increase (Mulkerrins et al., 2004).
(f) The accumulation of filamentous organisms prevents proper compaction and settling of sludges, hence reducing the quality of the effluent water (Wagner and Loy, 2002).
(g) Dissolved oxygen (DO) concentrations vary as per the treatment solution. The activated sludge systems need DO concentrations higher than 2 mg/L as they carry out processes like nitrification and carbon oxidation. On the other hand, the anaerobic zones of biological phosphorus removal systems must keep DO in the range 0.0–0.2 mg/L as substances like oxygen and nitrate interfere with biological phosphorus removal (Louzeiro et al., 2002).
(h) The process of nitrification is sensitive to pH and requires it to be 7.5–9, whereas denitrification happens at pH 7–8. Similarly, near neutral pH of around 7.2 is optimum for phosphorus removal, while acidic pH adversely affects this process (Mulkerrins et al., 2004).
(i) Heterotrophic Protists can help enhance nutrient cycling and carbon mineralization. This is done by them through either of the 3 hypothesized mechanisms, i.e., (a) their metabolisms cause release of N as ammonia or nitrate and P as phosphates which causes accelerated utilization of organic carbon by bacteria due to the need of maintaining their respective C: N: P ratio, (b) they produce amino acids, vitamins, and nucleotides that helps enhance bacterial activity as they are growth promoting in nature, and (c) they graze on bacteria causing inefficient (more carbon must be dissimilated to obtain the same amount of biomass as compared to a more efficient species) species that grow fast to get selected such that additional organic carbon resource is utilized in the process (Pogue and Gilbride, 2007).

#### Wastewater Treatment Using Up-Flow Anaerobic Sludge Blanket Reactor: A Bacterial Technology

**FIGURE 3:**
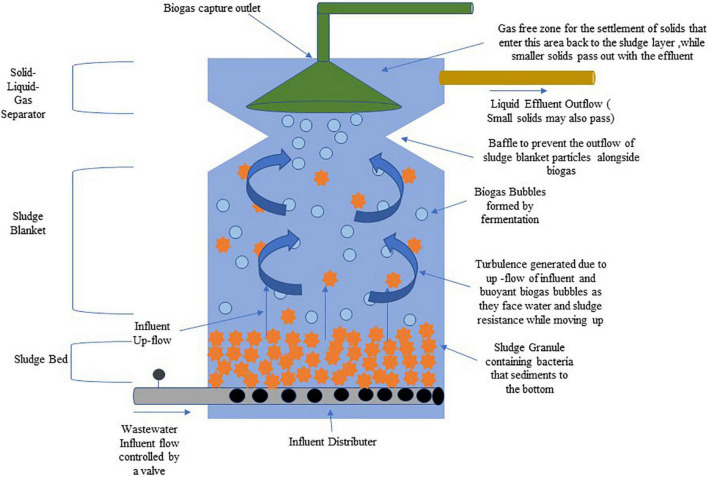
Schematic of an up-flow anaerobic sludge blanket (UASB) reactor system.

Anaerobic water treatment technologies have been used since the 19th century. Still, their treatment times are very slow if the water inflow volumes increased rapidly and had limited efficiency when compared to aerobic processes. This resulted in multiple modifications in their processes in the last part of the 20th century in terms of biomass accumulation, anaerobic metabolisms, effects of toxic compounds, and in the physiological interactions between different microbial species. These modifications made the anaerobic process an economically attractive alternative to the aerobic process (that is energy intensive) for the treatment of industrial wastewater in tropical to semi-tropical regions and even domestic wastewaters. One such treatment method is the up-flow anaerobic sludge blanket (UASB) process that Lettinga and coworkers developed in the 1970s (Bal and Dhagat, 2001). It is optimally used in high strength organic wastewater treatment as it produces high biomass concentrations and has a rich microbial diversity. This means that large volumes of organic wastes can be treated in compact reactors. The treatment performance is dependent on the granulation process, which is the formation of dense, multi-species microbial communities which cumulatively degrade complex organic wastes (Liu et al., 2003). The UASB reactor is of the vertical flow type that operates best in the mesophilic/thermophilic temperature range, with a solid, liquid, and gas phase separator that helps in the separation of biogas produced and helps return the dispersed sludge to sludge layer. Its operation and maintenance costs are 30% less than that required for the activated sludge process. The COD removal efficiencies for a UASB treating acidified food waste were recorded at 93% along with volatile fatty acid removal rates of 77–79% (Nnaji, 2014). Some advantages of using this treatment strategy include effective handling of organic shock loads, reduced CO_2_ emissions, and biosolid waste generation in comparison to the aerobic process. Disadvantages are that pathogen removal is only partial and there is a need for post treatment due to incomplete nutrients removal by it (Latif et al., 2011). Figure 3 depicts the scheme of an Up-flow Anaerobic Sludge Blanket (UASB) reactor system.

## The Integrated Bacterial-Microalgal Approach for Treatment of Wastewater

Bacterial–microalgal approach is converging point of interest in industrial areas in the urge of improving the ecological status of the water resources, in particular for minimizing the phosphorus and nitrogen content in wastewater effluents. The ability of microalgae to utilize the organic and inorganic carbon, as well as inorganic nitrogen (N) and phosphorus (P) in wastewater for their growth, with the desired results of a reduction in the concentration of these substances in the water demands application of microalgae (Wollmann et al., 2019). Grady et al. (2011) estimated that the organic material of phytoplankton biomass fabricated from the discharge of 1 kg of P can exert 100 kg of O_2_ demand, while that produced from the discharge of 1 kg of N can exert 14 kg of O_2_ demand, due to this hypoxic or anoxic conditions species diversity is lost and overall functionality of aquatic ecosystem influenced. Threshold concentration of the range between 0.21–1.2 mg L^–1^ and 0.01–0.1 mg L^–1^ of total nitrogen (TN) and total phosphorus (TP), respectively, responsible for causing the eutrophication, was evaluated by Chambers et al. (2012).

Mostly, P is considered as the rate limiting nutrient for phytoplankton growth; hence, for mitigating the eutrophication, there is a need to reduce the input of Phosphorous into the receiving systems (Hendriks and Langeveld, 2017). For example, in Denmark, a TP effluent concentration of 0.3 mg L^–1^ is applied to all municipal treatment facilities, whereas in Sweden a 90% reduction is required (compared to 80% reduction in relation to the load of the influent stated by the UWTD). The complex removal process of the N and P requires a separate environment which requires high energy which substantially results in the increases of overall cost of the treatment (Grady et al., 2011). The sustainable alternative to energy intensive and conventional biological treatment processes which is environmentally friendly as well includes the eukaryotic algae and cyanobacteria (Singh et al., 2015). Almomani et al. (2019) stated that the use of microalgae in wastewater treatment is a cost effective and feasible method for bio-fixation of CO_2_, in addition to being a renewable source for biomass. Along with the ability of microalgae to utilize the N and P, another advantage of using the microalgae into wastewater treatment is the generation of the oxygen by the process of photosynthesis. The microalgae have the ability to utilize the organic nitrogen and the inorganic nitrogen if available in the form of ammonium/ammonia as nitrite and nitrates.

**FIGURE 4:**
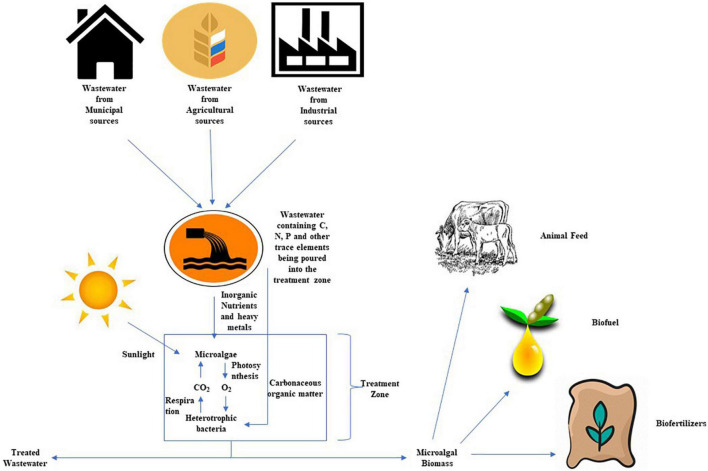
Integrating algae and bacteria for the degradation of wastewater contaminants.

Further, the added benefit of using integrated approach has been elucidated by Sturm and Lamer (2011) and Gouveia et al. (2016). According to authors, in the case of the wastewater treatment by an algal-bacterial co-culture approach, we need not to switch between the different operating environments to facilitate inorganic N and P removal, it just requires the single stage treatment, which in result reduces the complexity of the treatment process (Figure 4). Microalgae assimilate ammonia (NH_3_) and phosphate (PO_4_) directly and in concert for cell growth and metabolic function (Falkowski and Raven, 2007; Borowitzka et al., 2016). As the majority of N is being assimilated instead of being converted into oxides of nitrogen, the microalgae mediated treatment process releases lesser greenhouse gas than the conventional treatment. There is a negligible emission of Nitrogen Oxides using microalgal association in the wastewater treatment process (Fagerstone et al., 2011; Guieysse et al., 2013). Based on the analysis of Alcántara et al. (2015), a microalgae wastewater treatment process is estimated to have an emission factor of 0.0047% g N_2_O-N g^–1^ N-input. A number of studies which were done using microalgal species on different wastewater types include municipal, agricultural, brewery, refinery, and industrial effluents with different efficiencies, and this investigation into the biological removal of carbonaceous, nitrogenous, and phosphorus has been evaluated (Cai et al., 2013; Gentili, 2014; Chiu et al., 2015).

Ji et al. (2013) and Prandini et al. (2016) have demonstrated that the strain *Scenedesmus obliquus* successfully removed nutrients (carbon, N and P) from piggery wastewater. Kothari et al. (2012) showed that *Chlorella pyrenoidosa* after wastewater acclimatization (at a 75% concentration optimum) is seen to successfully grow in the dairy wastewater effluent while also reducing mineral concentrations and chemical characters like TS (total solids) and Hardness. Some species of *Chlorella* such as *C. vulgaris* have also been reported using N and P from the municipal wastewater effluent at the primary stage of the treatment (PO_4_^3–^-P: 8–3 mg L^–1^; NH_4_^+^: 119–37 mg L^–1^), secondary stage (PO_4_^3–^-P: 6.1–0.5 mg L^–1^; NH_4_^+^-N: 6.9–0.8 mg L^–1^), and from centrate (TP: 215–40 mg L^–1^; TN: 116–12 mg L^–1^, Gouveia et al., 2016). Choi (2016) have proved that the reported 88% biological oxygen demand (BOD), 82% TN, and 54% TP have been successfully removed from the initial concentrations in the brewery effluent using *C. vulgaris*. Other micro-algae species examined for their bioremediation potential include *Chlamydomonas* sp., *Nannochloropsis*sp., *Dunaliella* sp., *Spirulina* sp., and *Botryococcu* sp. (Cuellar-Bermudez et al., 2015; Gonçalves et al., 2017). The amount of nutrients utilized by the microalgae is closely associated with their growth (Xin et al., 2010; Al Ketife et al., 2017) limiting the supply of these nutrients can also limit or reduce their growth. So, optimal ratio of nutrients that is essential for the microalgal must be present in the cultivation media to ensure the optimal removal of N and P. The traces of the micronutrient which are essential such as the calcium, magnesium, potassium, manganese, silica, zinc, iron, and other are generally present in the abundant amount in the wastewater (Falkowski and Raven, 2007; Borowitzka and Moheimani, 2013).

The microalgal removal efficiencies differ with the different concentration of the C, N, and P. *Chlorella* sp. had an improved efficiency in inorganic N and P removal associated with the higher average specific growth rate in PSW (poultry slaughterhouse water) as compared to STE (secondary treatment effluent, Wang et al., 2010). In total, 68.5% of TN and 90.6% of TP is the removal capacity of this species microalgae from the PSW, and from the STE the removal capacity is 50.8% TN and 4.96% TP. Furthermore, a decline of 56.5% was observed in the level of COD from the PSW, and an increase of 22.7% was recorded in the STE which signifies that the microalgae produces the oxidizable carbon matter. Various studies showed that in respect to the bioavailable (N and P) the ability of microalgae to grow and treat wastewater deviates from the canonical N and P stoichiometry of freshwater microalgae. For example, a lower residual concentration when the synthetic water is treated with the microalgae *C. vulgaris* after establishing an optimum *N*:*P* ratio (Klausmeier et al., 2004; Xin et al., 2010; Borowitzka et al., 2016). A decrease from 5.1 to 2 mg L^–1^ was noticed in this study when the *N*:*P* ratio was increased from 4:1 to 8:1 and a significant decline in removal efficiency occurred as a result of the increasing ratio. Al Ketife et al. (2017) showed a slightly higher optimal ratio of N and P for a different *C. vulgaris* strain, with the complete removal of N and P which is achieved at a ratio of 10:1. Similarly, Arbib et al. (2017) examined that the ratio of N and P should be between 9:1 and 13:1 for the efficient removal of the nutrients for *S. obliquus* species. Number of studies on various culturing techniques have proved the success of incorporating microalgae in treating the PSW. According to Choi (2015) upto 96.38% of TN and 92.7% of TP was removed from Municipal Wastewater upon treating it in a microalgal bioreactor containing a *C. vulgaris* culture. Further, Ramos Tercero et al. (2014) and Almomani and Örmeci (2016) reported the efficiencies of 63.2% NH_4_^+^-N, 32.4% total dissolved P, and 64.9% removal from the PSW using a native microalgal species. These removed nutrients are used for algal biomass generation, and Table 2 shows the rate at which this happens in different algal species.

**TABLE 2:** Showing the rate at which different microalgal species grow by assimilating nutrients into their biomass under specific condition sets.

Microalgae	Taxonomic order	Biomass productivity as per studies (kg/m^3^/day)	References
*Chlorella vulgaris*	Chlorellales	0.35	Blair et al., 2014
*Chlorella sorokiniana*	Chlorellales	0.85	Ugwu et al., 2007
*Chlamydomonas* sp. (YG04)	Chlamydomonadales	0.0552	Shekh et al., 2016
*Coelastrum microporum*	Sphaeropleales	0.044	Hussain et al., 2020
*Oocystis* sp.	Chlorellales	0.02524	Mohammadi et al., 2018
*Chlorella zofingiensis*	Chlorellales	0.29616 ± 0.01916	Li et al., 2019
*Scenedesmus obliquus*	Sphaeropleales	0.29250	Ho et al., 2010
*Scenedesmus dimorphus*	Sphaeropleales	0.09	Chng et al., 2017
*Mucidosphaerium pulchellum*	Chlorellales	0.1889 ± 0.010	Mehrabadi et al., 2017

Algal based wastewater treatment is more sustainable compared to bacterial based wastewater treatment (Kohlheb et al., 2020). This has been proved by HRAP which is more beneficial than activated sludge based sequencing batch reactors (SBR) both environmentally as well as economically. This is because the total electricity consumption of HRAP is only 22% of the electricity consumption of SBR. Also at discount rates of 0.25 and 3% the life cycle costs of HRAP to SBR was found to be 0.182 €/m^3^ vs. 0.258 €/m^3^ and 0.125 €/m^3^ vs. 0.167 €/m^3^, respectively. Also, the global warming potential in terms of net CO_2_ production for HRAP to SBR was 146.27 vs. 458.27 × 10^–3^ kg CO_2_ equiv./m^3^, respectively. Similarly, eutrophication potential in terms of phosphate release was 126.14 vs. 158.01 × 10^−6^ kg PO_4_ equiv./m^3^, respectively.

### Merits and Demerits of the Integrated Bacterial-Microalgal Approach for Treatment of Wastewater

On one hand, the integrated approach has some merits to its usability (Katam and Bhattacharyya, 2021). These include the (a) ability for single step removal of nutrients and organic carbon, (b) such systems have lower requirement for mechanical aeration, due to which their carbon footprint is less, (c) resourceful biomass generated in the form of biomass that can be used for the synthesis of biodiesel or biogas, (d) lower quantities of sludge generated in comparison to typical treatment technologies that use bacteria, and (e) the wastewater effluent produced is disinfected from pathogens within the system due to which additional chemicals are not required later. On the other hand, they have found certain demerits of this approach too, which are (a) light dependency of microalgal growth which is not true for pure activated sludge process, (b) high pH of medium generated due to photosynthesis by microalgae that negatively impacts the bacterial consortium associated with the activated sludge process, (c) the slow growth rate of microalgae which leads to higher Solid Retention Time (SRT) than what is required by heterotrophic organisms, and (d) difficulty associated with the separation of microalgal biomass from the mixed liquor so that lipids and pigment can be extracted. Other than these, treatment of the wastewater using microalgae faces more challenges which includes dealing with the large volumes of wastewater that needs to be treated. Hoh et al. (2016) stated that the various microalgae cultivation processes need to be reviewed to come out with (a) optimal microalgal productivity, (b) for ensuring the higher effectiveness of the nutrient removal, and (c) to deal with large volumes. The wastewater treatment process is also largely affected by the design and configuration of the reactor controlling measures like light and temperature which have influence on the growth of microalgae (Mata et al., 2010). Christenson and Sims (2011) have classified the different microalgal techniques broadly into two categories either suspended or immobilized systems. The two systems are further classified as either open or closed systems. Hoh et al. (2016) stated that the different types of bioreactors are considerably important when looking at the efficiency of the wastewater treatment and the biomass productivity. As per the Swedish EPA (2008), in anticipation of the getting the lower set standards, the performance of the treatment process should be able to meet the current required effluent concentrations which has been set by the UWTD (urban wastewater treatment directive).

Smaller the reactor system shorter will be its HRT (hydraulic retention time), which makes it better for saving capital costs and helps in optimizing the surface area requirement (Metcalf and Eddy, 2003; Ruiz et al., 2013). Several studies suggested that out of immobilized and suspended cultivation systems, a shorter treatment duration is observed in PBR (photobioreactor) suspended systems, with higher N and P removal efficiency despite the various operating parameters like biomass inoculation concentration, temperature, and irradiance. In PBR suspended systems, an average 87.3% N and 82.9% P removal efficiency was achieved, within an average of 3.1 days (or HRT, Choi, 2015). Some current evidence shows that the integration of microalgae with bacteria for wastewater treatment is a technologically and environmentally feasible option. When microalgae is used as the option for the removal of nitrogenous, phosphorous, carbonaceous material, it works at a lower footprint in terms of the energy consumption and greenhouse gas generation as compared with the conventional wastewater treatment process.

In current times, decentralization of wastewater treatment has become necessary as centralized wastewater treatment plants (WWTPs) have not been able to keep up with the pressure of catering to the ever increasing population in urban areas. They are also unable to expand their current capacities as most of them are located in cities where there is a space constraint. The application of the integrated approach in addressing this problem has been studied by Katam and Bhattacharyya (2021). According to authors, the application of algal biofilms to an already existing ASP (activated sludge process) based DWWTP (decentralized wastewater treatment plant) in Hyderabad, India leads to a significant enhancement of carbon and nutrient removal from the influent wastewater inside a single reactor. For this purpose, the algae have been provided with the optimal light intensity, photoperiod, and immobilized using a suitable technique (Katam and Bhattacharyya, 2021).

### Factors Influencing Bacteria-Microalgae Wastewater Treatment

Bacteria, industrial contaminants, pH, light, and temperature are some biotic and abiotic factors that influence the course of action of the microalgal wastewater treatment. Bacteria is an important factor whose presence has a positive influence on the microalgae-bacteria wastewater treatment process. Bacteria is mandatory and essential for microalgae as through its heterotrophic metabolism it may support the growth of the microalgae by providing CO_2_ mineralizing it into a form of inorganic compounds which can be directly taken up by the microalgae. In return, the microalgae provide O_2_ generated during the process of photosynthesis required by the heterotrophic bacteria for degrading the organic matter and again required back by the microalgae during dark respiration (Falkowski and Raven, 2007). Su et al. (2011) reported that during the treatment process of primary sewage waste with the microalgal consortium resulted in the enrichment of certain bacterial species which are dominated by the members of the classes Bacteroidia (50%), Flavobacteria (25%), Betaproteobacteria (12.5%), and Gammaproteobacteria. In a subsequent study, for investigating the removal efficiency of contaminants from PSW, Su et al. (2011) inoculated the sludge with the different microalgal ratios and found fluctuations in the occurrence of bacterial community composition among the treatments of different inoculation ratios. In contrast, other studies (Schumacher et al., 2003; Ansa et al., 2011; Sousa, 2013) have mentioned that the bacteria and microalgae have adverse effects on each other *via* some abiotic factor. Bacterial activity was highly affected by an increase in the pH and dissolved O_2_ in the algal cultures. Another study shows a strong interrelation between the abundance of bacteria (heterotrophic bacteria) and pH which shows the negative impact of inactivation of bacteria with increasing pH (Awuah et al., 2001; Ansa et al., 2011, Ansa et al., 2012). Temperature also has a great influence on the microalgae-bacteria wastewater treatment as it is highly necessary for normal functioning of the microalgae as the energy captured by them from the light help in driving the O_2_ evaluation process to generate the ATP and the reducing agents that are required for fixing CO_2_ into organic carbon (Falkowski and Raven, 2007; Williams and Laurens, 2010).

Proper maintenance of microalgal growth promoting environment is necessary as the nutrient deficient condition might induce resource competition between the existing microbial community and exogenous microalgae (Grover, 2000). Certain studies have described a saturation point below that the rate of photosynthetic activity is proportional to the irradiance intensities, receptor system become damaged resulting into photo-inhibitions if the intensities above the saturation point is illuminated (Falkowski and Raven, 2007; Williams and Laurens, 2010). This illumination intensity may vary with respect to the microalgal species and temperature. Generally, the illumination saturation point are found to fall between 200 and 400 μE m^–2^ s^–1^ for the freshwater microalgae (Muñoz and Guieysse, 2006; Cheirsilp and Torpee, 2012; Singh and Singh, 2015). Therefore, maintaining the culture system at or below the saturation point is another important and practical part in order to avoid excessive light utilization by algae and also to avoid over expenditure of energy. The illumination period and the intensities have a link to removal efficiency of the inorganic N and P from the wastewater. Lee et al. (2015) reported a reduction in the capacity, when the removal of N and P from the wastewater is allowed with the microalgal-bacterial consortium under prolonged dark conditions.

## Conclusion and Future Perspectives

In India and other developing countries, 50–80% of the wastewater is discharged untreated or partially treated. The amount of wastewater is increasing day by day in tune with urbanization and global population increase. The conventional sewage treatment systems like activated sludge fail to reduce nutrient concentration of wastewaters to acceptable levels for preventing rapid rate of eutrophication in the vicinity of sewage discharge sites. The addition of microalgae to the sewage treatment has multiple applications such as (a) removal of excess inorganic nitrogen, phosphorus, and heavy metals, (b) non-proliferation of pathogenic bacteria, (c) microalgae mediated CO_2_ sequestration, and (d) use of microalgae for biofuel production. The integrated bacterial-microalgal approach proves to be even more promising as the heterotrophic bacteria have potential to degrade the organic matter in the absence of aerated O_2_, as O_2_ is provided by the microalgal photosynthesis and similarly the need for CO_2_ sparging is also eliminated as this is produced by the bacterial respiration. Therefore, the integrated approach of bacteria-microalgae wastewater treatment would require single stage treatment reducing the complexity of the treatment process. Heterotrophic bacteria utilize the organic compounds of the waste water and produce CO_2_, whereas the microalgae in turn utilize this CO_2_ to produce carbohydrates and O_2_ through photosynthesis, of which the former is needed for biomass production, while the latter serves as the terminal electron acceptor for aerobic respiration in bacteria. So the application of heterotrophic bacteria and autotrophic protists (algae) has the potential to complement each other, making them easy to handle and safe to be used by people. This cyclical, almost self-reliant water treatment method with the added benefit of microalgal biomass could provide the right balance between expenditure and the access to clean water to the citizens, where wastewater treatment is rare, environment protection standards are not stringent, and water borne diseases are common. Being eco-friendly, safe to handle and cost effective such a system can be applied in a decentralized manner at source rather than the conventional STPs that need a centralized large scale plant. In tune with increasing amount of waste water load and aiming to have good biomass yields optimizing the relative proportion of microalgae and bacterial consortium further research is needed to be done in this area. This requires critical understanding of interactions between the necessary bacterial species and microalgae to stabilize treatment processes and their growth promoting factors, like pH level, temperature, and nutrient concentration. Ways to monitor and provide a continuous supply of nutrients and light must be ensured (mainly in closed systems) for optimal performance. In wastewater discharge zone meiobenthos are observed in high numbers, and they are known to utilize organic debris settled at the bottom. The possibility of adding meiobenthic fauna (benthic metazoans that can pass through a 500 μm filter, but which are retained on a 40 μm one) could also be evaluated alongside bacteria and microalgae. This is attributed to their ability to feed on particulate organic matter, thus hastening the process of degradation of organics. They could also act as the predators of the bacteria that make biofilms in the filters used for physical treatment of wastewater, hence providing a natural alternative for cleaning them. The bacterial biomass developed during the treatment process could also be diverted toward these meiobenthos, hence reducing their numbers in the effluent naturally rather than relying on physical and chemical disinfection processes like UV or chlorine treatment. Still, the inclusion of such disinfection processes may provide superior performance.

## Author Contributions

MM, KK, VV, and RD wrote the original draft of the manuscript. RD supervised the manuscript. RD and MM conceived the concept and designed of the manuscript. RK gave critical feedback and edited the manuscript. H-UD and J-SH contributed to the revision of the manuscript. All authors contributed to the article and approved the submitted version.

## Conflict of Interest

The authors declare that the research was conducted in the absence of any commercial or financial relationships that could be construed as a potential conflict of interest.

## Publisher’s Note

All claims expressed in this article are solely those of the authors and do not necessarily represent those of their affiliated organizations, or those of the publisher, the editors and the reviewers. Any product that may be evaluated in this article, or claim that may be made by its manufacturer, is not guaranteed or endorsed by the publisher.

## Abbreviations

NADPH: nicotinamide adenine dinucleotide phosphate(reduced)
ATP: adenosine triphosphate
PMF: proton motive force
PAR: photosynthetically active radiation
GOGAT: glutamine oxoglutarate aminotransferase
NADP: nicotinamide adenine dinucleotide phosphate
DNA: deoxyribonucleic acid
RNA: ribonucleic acid
ADP: Adenosine diphosphate
TCA: tricarboxylic acid cycle
Eutrophication: when water body becomes excessively enriched with nutrients
TP: total phosphorus
TN: total nitrogen
PSW: poultry slaughterhouse water
STE: secondary treatment effluent
UWTD: urban wastewater treatment directive
HRT: hydraulic retention Time
PBR: photobioreactor.
